# Mammography screening and incidence of ductal carcinoma *in situ* of the breast in Italy: an age-period-cohort analysis

**DOI:** 10.1093/ije/dyaf102

**Published:** 2025-06-24

**Authors:** Lauro Bucchi, Silvia Mancini, Annibale Biggeri, Rosa Vattiato, Orietta Giuliani, Alessandra Ravaioli, Flavia Baldacchini, Federica Zamagni, Fabio Falcini

**Affiliations:** Emilia-Romagna Cancer Registry, Romagna Cancer Institute, IRCCS Istituto Romagnolo per lo Studio dei Tumori (IRST) Dino Amadori, Meldola, Forlì, Italy; Emilia-Romagna Cancer Registry, Romagna Cancer Institute, IRCCS Istituto Romagnolo per lo Studio dei Tumori (IRST) Dino Amadori, Meldola, Forlì, Italy; Unit of Biostatistics, Epidemiology and Public Health, Department of Cardiac, Thoracic, Vascular Sciences and Public Health, University of Padua, Padua, Italy; Emilia-Romagna Cancer Registry, Romagna Cancer Institute, IRCCS Istituto Romagnolo per lo Studio dei Tumori (IRST) Dino Amadori, Meldola, Forlì, Italy; Emilia-Romagna Cancer Registry, Romagna Cancer Institute, IRCCS Istituto Romagnolo per lo Studio dei Tumori (IRST) Dino Amadori, Meldola, Forlì, Italy; Emilia-Romagna Cancer Registry, Romagna Cancer Institute, IRCCS Istituto Romagnolo per lo Studio dei Tumori (IRST) Dino Amadori, Meldola, Forlì, Italy; Emilia-Romagna Cancer Registry, Romagna Cancer Institute, IRCCS Istituto Romagnolo per lo Studio dei Tumori (IRST) Dino Amadori, Meldola, Forlì, Italy; Emilia-Romagna Cancer Registry, Romagna Cancer Institute, IRCCS Istituto Romagnolo per lo Studio dei Tumori (IRST) Dino Amadori, Meldola, Forlì, Italy; Emilia-Romagna Cancer Registry, Romagna Cancer Institute, IRCCS Istituto Romagnolo per lo Studio dei Tumori (IRST) Dino Amadori, Meldola, Forlì, Italy; Cancer Prevention Unit, Local Health Authority, Forlì, Italy

**Keywords:** breast neoplasms, ductal carcinoma *in situ*, mass screening, mammography, incidence, age-period-cohort analysis

## Abstract

**Background:**

The increasing incidence of ductal carcinoma *in situ* (DCIS) of the breast is attributed to mammography screening, but the trend has so far been evaluated only descriptively.

**Methods:**

We report an age-period-cohort modelling analysis of the incidence trend (1992–2017) observed among screening-aged women living in a region of northern Italy, where a mammography screening programme was implemented in 1996–98 (age 50–69 years) and 2010–14 (age 45–49 and 70–74 years).

**Results:**

The incidence of DCIS increased by an annual average of 9.1% (age 60–64 years) to 14.9% (age 70–74 years). The incidence peak followed a complex age-time pattern indicating an interaction between age and period, which suggested a cohort effect explained by the screening programme. In the age-period-cohort analysis, the birth cohort had a 2-fold effect. The nonlinear effect consisted of an increase in incidence for the generations of 1933–42 and 1943–52, targeted by screening since 1997, and of a second increase for the cohort of 1963–72, first invited in 2010. Taking into account the early excess incidence due to the introduction of the screening programme, the linear effect consisted of an annual 4.0% increase in the risk of DCIS for all successive birth cohorts or calendar periods, which was only partially attributable to the programme.

**Conclusions:**

The increase in incidence rates resulted from an increased detection of DCIS through the screening programme and from an uptrend in the risk of diagnosis that can be attributed either to long-term changes in diagnostic scrutiny independent of the programme or to an increased exposure to risk factors.

Key Messages

- The increasing incidence of ductal carcinoma *in situ* (DCIS) of the breast is attributed to mammography screening, but the trend has so far been evaluated only descriptively.
- This is the first age-period-cohort modelling analysis of the incidence trend of DCIS before and after the implementation of an organised screening programme (northern Italy, 1992–2017).
- The observed strong increase in DCIS rates resulted from an increased detection through the screening programme and from an uptrend in the risk of diagnosis that can be attributed either to long-term changes in diagnostic scrutiny independent of the programme or to an increased exposure to risk factors.

## Introduction

Ductal carcinoma *in situ* (DCIS) of the breast is at the centre of the dispute about the benefit-to-harm ratio in mammography screening [1, 2]. The diffusion of mammography use is generally indicated as the cause of the increased incidence of DCIS and, by implication, the disease is considered a major driver of overdiagnosis [3, 4]. A supporting evidence is that the detection of DCIS has not yielded clear benefits, in that invasive breast cancer (BC) rates have continued to rise [1, 5, 6].

The huge variation in the incidence trend of DCIS that has been observed following the introduction of mammography screening [7], from nonsignificant [8] or moderate [7, 9] impacts to about 5-fold [1] and even larger [5] increases, is not a strong argument against a causal relationship between screening practices and the detection of DCIS. The screening programmes, indeed, may differ substantially as to uptake rate, mammography sensitivity, and rapidity and completeness of introduction of digital mammography, high-resolution ultrasound, and stereotactic breast biopsy [10, 11]. The impact of screening on the incidence trend of DCIS may also be influenced by differences in the diagnostic workup of women with abnormal mammography results, for example in the criteria for pathologic diagnosis and particularly in the policy-determined threshold for the pathologic identification of lesions as DCIS, and by differences in the management of the disease. The option of no surgery [12], indeed, may have an effect on the completeness of case ascertainment of cancer registries. Finally, the varying degree of completeness of cancer registration over time and space may confound the apparent relationship between the introduction of screening programmes and DCIS incidence.

The published studies, however, have evaluated the incidence trend of DCIS using a purely descriptive approach [1, 5–8, 13–15] with only anecdotal exceptions [9]. Consequently, the literature has left unanswered the question of whether the diffusion of mammography use is the only cause for the increasing incidence of the disease. An uptrend in the risk of invasive BC in women aged 20–39 years has been reported from many developed countries, including Italy, in the past three decades [16]. In the same years, disease-specific mortality rates have decreased minimally for Italian women aged 20–49 years while dropping to a substantial extent among older ones [17]. Overall, this age pattern is compatible with, though not proof of, a birth-cohort-dependent increase in the risk of invasive BC. This provides the rationale for investigating the incidence trend of DCIS with an age-period-cohort (APC) approach, in order to disentangle the impact of exposure to screening from the underlying secular trend (if any). We have carried out a study of this design to characterise the trend (1992–2017) in the incidence rates of DCIS among screening-aged women living in Romagna (northern Italy) before and after the implementation of an organised mammography screening programme.

## Methods

### Set-up of the screening programme by age, calendar year, and birth cohort

The screening programme is part of the Emilia-Romagna regional screening programme [18–20]. In the subregion of Romagna, the first screen was implemented in 1996, 1997 (for the greater part), and 1998. The target population included resident women aged 50–69 years. The screening test was a biennial, two-view, film-screen, double-read mammography. The transition to digital mammography began during the first decade of 2000s and was completed in 2010–11 [21]. During the entire study period, digital breast tomosynthesis was not yet in use as a screening test.

**Figure 1.**
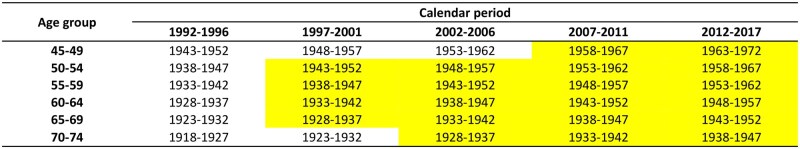
Lexis diagram showing the 5-year calendar period-specific (6 years for the period 2012–17), the 5-year age groups, and the 10-year birth cohorts. Highlighted in yellow are the birth cohorts invited to the screening programme in specific age groups and calendar periods. Romagna (northern Italy), 1992–2017.

In 2010, the target age was extended to include women aged 45–49 (annual mammography) and 70–74 (biennial mammography) years. Their enrolment was completed in 5 years (2010–14) and 2 years (2010–11), respectively [22]. Figure 1 shows which cohorts were invited, at which age and when. The current target population aged 45–74 years comprises 278 000 women.

### Data

The local cancer registry [23] follows the International Agency for Research on Cancer (IARC) coding rules, which also apply to multiple primary cancers [24]. When a DCIS is followed by an invasive BC with the same International Classification of Diseases for Oncology (ICD-O)-3 morphology code, only the invasive primary is retained in the ‘clean’ database in order to generate standard incidence statistics.

Consequently, the study data were retrieved from the ‘service’ database, a specially configured additional system that is used for internal purposes and retains all types of information collected, including the notifications of DCIS followed by the notification of an invasive BC. The completeness of data extraction from this source was manually tested on the records of a random sample of 2600 breast neoplasms. Given their nonstandard nature, the data were quality-assured according to the usual procedures [25].

We excluded those patients with any of the following: history of invasive BC prior to DCIS diagnosis; simultaneous diagnosis of invasive BC and DCIS [7]; *in situ* BC other than DCIS (ICD-O-3 topography code C50 and morphology codes 8050, 8140, 8200, 8201, 8211, 8230, 8246, 8260, 8343, 8401, 8480, 8481, 8490, 8500, 8501, 8503, 8504, 8507, 8508, 8521, 8522, 8523, 8543, or 8550) [6]; age below 45 years and above 74 years; sarcoma; occult primary cancer; no histologic confirmation; and notification by death certificate and autopsy report only. Overall, the study was based on 2426 DCIS cases registered in the years 1992–2017.

### Statistical methods

The population data used for calculating the incidence rates of DCIS were obtained from a public source of demographic data available online from the website of the Italian National Statistics Institute [26].

The strategy of statistical analysis was as follows. In the first place, we plotted the curves of annual age-specific DCIS incidence rates. A locally weighted scatterplot smoothing (LOWESS) [27] was used to add fitted curves to the observed values.

Then, we assessed the temporal trend. The estimated average annual percent change (EAAPC), with 95% confidence interval (CI), was calculated by fitting a weighted generalised linear regression model for the natural logarithm of the age-standardised rate and year as a linear trend, with a Gaussian distribution and identity link function.

Finally, we performed the APC analysis of the incidence trend [28, 29]. The incidence data were tabulated into six 5-year age groups, five 5-year calendar periods (6 years for the period 2012–17), and 10 overlapping 10-year birth cohorts [30]. The age-specific rates were plotted against the period and the cohort. We fitted five Poisson regression models according to the model-building approach [28, 29]. The model goodness-of-fit was assessed using residual deviance statistics. The models were compared using conditional likelihood ratio tests between hierarchically nested models and the Akaike information criterion [31].

To overcome the problem of nonidentifiability associated with the APC modelling, we first constrained the linear component of the period effect to have zero slope, thus assuming that the linear changes in incidence were due to cohort-related factors. Therefore, the linear trend (drift) was allocated to the cohort. The cohort of women born between 1948 and 1957, hereby referred to as the cohort of 1948–57, was the median cohort with respect to the number of cases and was used as a reference. The linear and nonlinear cohort effects were expressed as incidence rate ratios (IRRs). Cohort effect estimates were modelled using restricted cubic spline functions with three degrees of freedom. Pointwise CIs were computed.

As a final step of the APC analysis, we included a dummy variable for the presence/absence of the screening programme (Fig. 1, cells highlighted in yellow) and a term for the cumulative exposure to screening (Supplementary Fig. S1, available at *IJE* online). The rationale was that the screening programme acts as an exposure, which varies according to the period and the age group. Consequently, the screening programme created a cohort effect. Repeating the APC analysis after including exposure to screening as a covariate was expected to explain this effect.

The data analysis was made using the Stata statistical package, version 15.1 (Stata Corporation, College Station, Texas) and the APCfit Stata command [32].

## Results

### Trend in annual incidence rates

**Figure 2.**
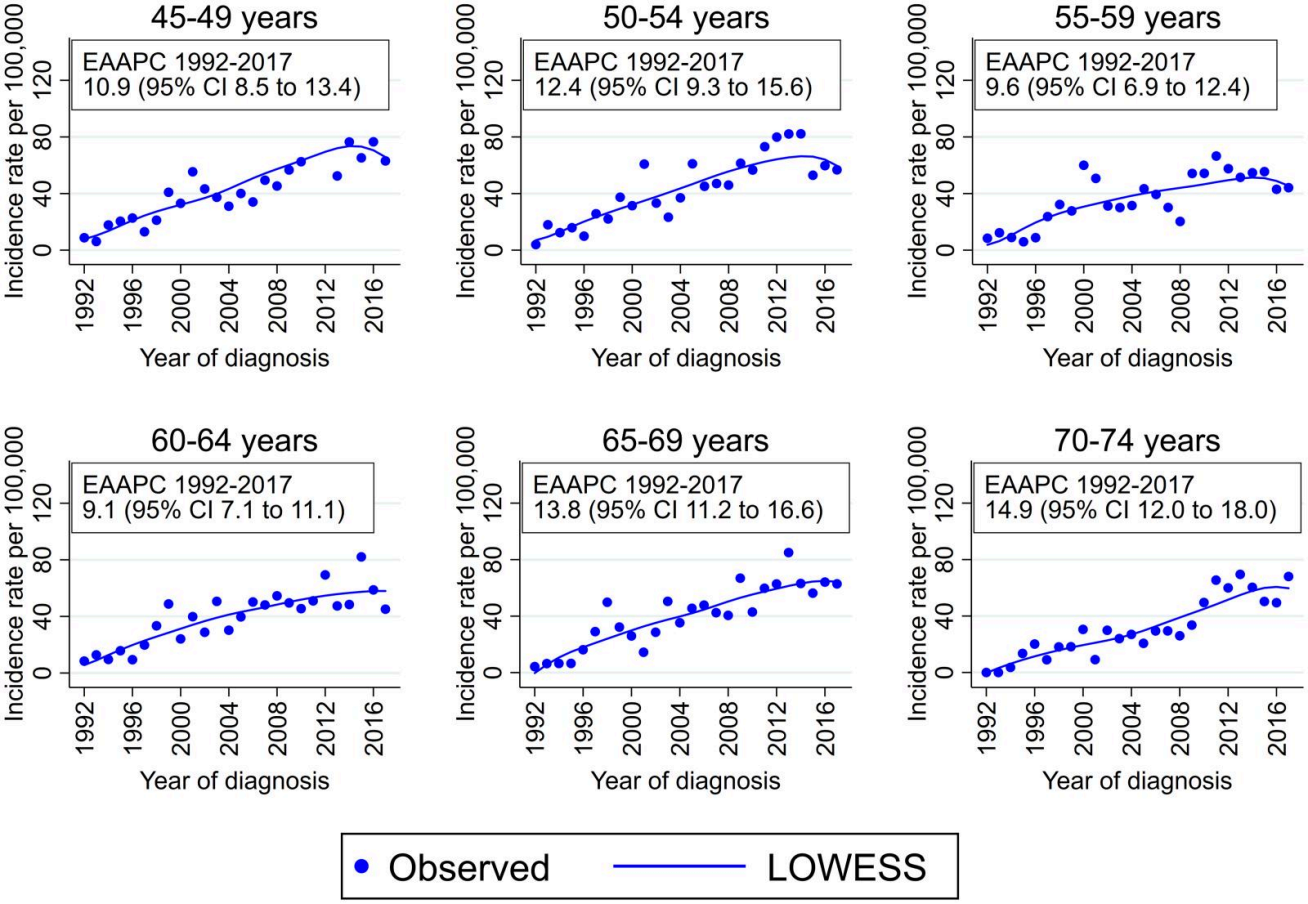
Observed and smoothed (LOWESS) curves of annual incidence rates of ductal carcinoma *in situ* of the breast per 100 000 women, by 5-year age groups. Romagna (northern Italy), 1992–2017.

The smoothed curves of annual incidence rates of DCIS between 1992 and 2017, by 5-year age groups, are shown in Fig. 2. A generalised and steep increase was observed, with the EAAPC varying between 9.1% (age 60–64 years) and 14.9% (age 70–74 years).

### Incidence rates by age and calendar period

**Figure 3.**
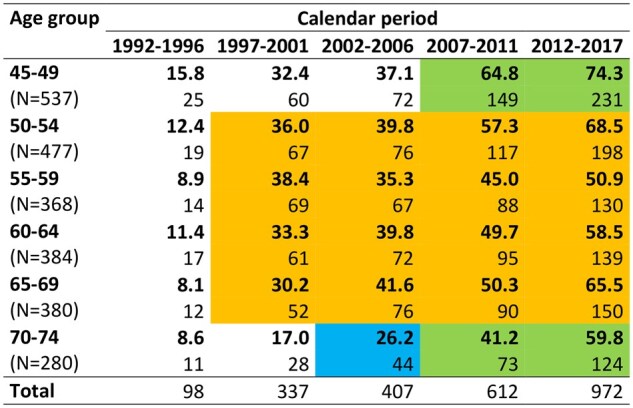
Incidence rates of ductal carcinoma *in situ* of the breast per 100 000 women and number of cases, by 5-year age groups and 5-year calendar periods (6 years for the period 2012–17). The cells highlighted in orange contain the data for women aged 50–69 years, invited to the screening programme since 1997. The cells highlighted in green contain the data for women aged 45–49 and 70–74 years, invited since 2010. The cell highlighted in blue contains the data for the cohort of women invited once in 1997, when they were in the age range 50–69 years. The remaining cells contain the data for women never invited. Romagna (northern Italy), 1992–2017.

The incidence rates by 5-year age groups and 5-year calendar periods are shown in Fig. 3. In 1992–96, when no age group was targeted by the screening programme, the rates were manifold lower. With the increase observed thereafter, the IRR between the last and the first calendar period varied between 4.71 (95% CI: 3.12; 7.12) for women aged 45–49 years and 8.04 (95% CI: 4.47; 14.48) for women aged 65–69 years.

Figure 3 also reveals that the incidence peak followed a complex age-time pattern. The risk was highest at age 45–49 years in the first period, at age 50–69 years in the subsequent two periods, and again at age 45–49 years between 2007 and 2017. Women aged 70–74 years exhibited generally lower rates, except for the years 2012–17. These data indicate an interaction between the age group and the calendar period, suggesting a cohort effect which could be explained by the screening programme (see below).

Supplementary Figure S2A, available at *IJE* online, shows the plots of calendar period-specific incidence rates by 5-year age groups. The risk increase over time was confirmed but with an acceleration in the years when the different age groups were invited to screening, particularly for women aged 50–69 years in 1997.

Supplementary Figure S2B, available at *IJE* online, shows the age-specific incidence rates by 5-year calendar periods. Before 1997, women aged 45–49 years had the highest rate. When the screening programme was implemented (1997–2001), the rates for the age group 50–69 years rose to levels comparable to those of younger women. In the last two periods, when the screening programme was extended to include younger and older women, the curves took a shape similar to the one of 1992–96, although at a higher level.

### Incidence rates by age and birth cohort

Supplementary Figure S3, available at *IJE* online, shows the plots of birth cohort-specific incidence rates by 5-year age groups. The risk showed a linear increase by cohort in the age groups not targeted by the screening programme until 2010 (45–49 and 70–74 years). In the other age groups, the risk increased for successive cohorts, but not in a linear fashion. The most rapid increases occurred at entry into the screening programme. This is case, for example, for women aged 55–59 years in the cohort of 1938–47.

**Figure 4.**
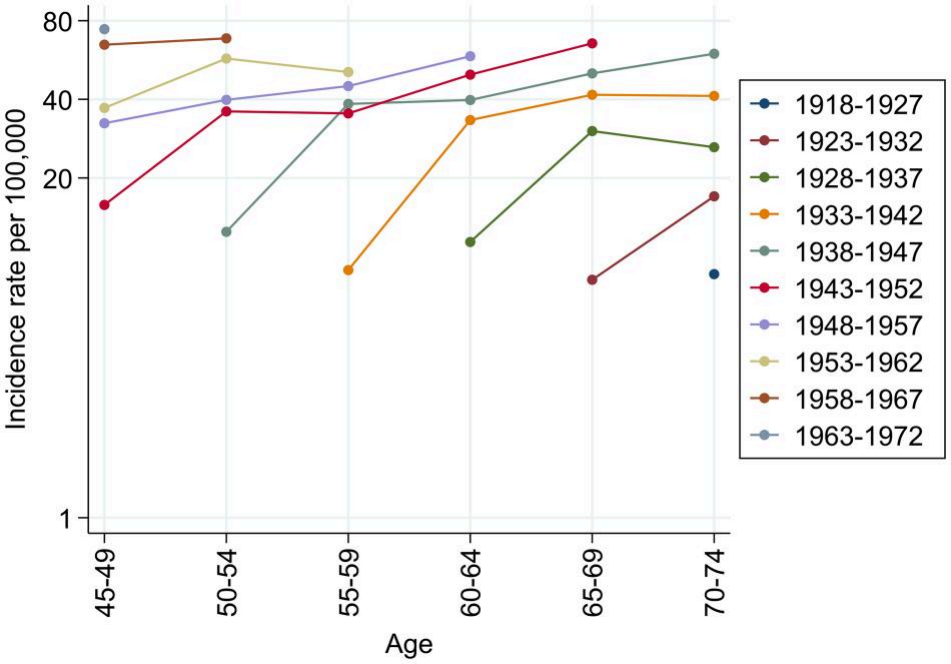
Plots of 5-year age group-specific incidence rates of ductal carcinoma *in situ* of the breast per 100 000 women, on a semilogarithmic scale, by 10-year birth cohorts. Romagna (northern Italy), 1992–2017.

Figure 4 shows the plots of age-specific rates according to birth cohort. The rates increased with increasing age. The most pronounced increases were observed at those ages, which varied depending on the cohort, when women entered the screening programme.

### APC modelling

**Figure 5.**
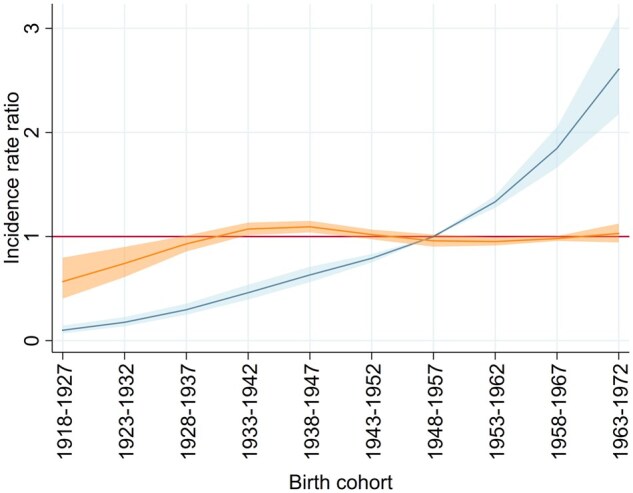
Cohort effect estimates from the age-period-cohort model fitted on incidence rates of ductal carcinoma *in situ* of the breast per 100 000 women. The cohort effect estimates (rate ratios) were obtained taking the birth cohort of 1948–57 as a reference (red line). The blue line (with shaded 95% confidence interval) indicates the pooled linear and nonlinear cohort effects. The orange line indicates the nonlinear cohort effect alone (with shaded 95% confidence interval). Romagna (northern Italy), 1992–2017.

In the APC analysis, the full APC model was the best-fitting one (Supplementary Table S1, available at *IJE* online). As shown in Fig. 5, the birth cohort showed a nonlinear effect. The cohort of 1948–57 was taken as a reference for the IRRs. The nonlinear effect consisted of a moderate increase in incidence for the generations born in 1933–42 and 1943–52, who have been targeted by screening since 1997, and of a second, less pronounced, increase for the cohort of 1963–72, who was first invited in 2010. The linear effect consisted of an increase in the risk of DCIS for all successive birth cohorts or calendar periods.

Subsequently, we included a dummy variable for the presence/absence of the screening programme in each cell of the Lexis diagram shown in Fig. 1. In this way, we took into account the early excess incidence due to the introduction of the screening programme. The age-period model provided the best fit to the data (Supplementary Table S2, available at *IJE* online). The nonlinear cohort effect was entirely explained by the implementation of the screening programme. We also found a linear increase in incidence of 4.0% (95% CI: 1.9%; 6.1%) per year, which can be interpreted either as a trend by cohort or as a trend by calendar period.

Finally, we augmented the above APC models by adding a term for the cumulative exposure to the screening programme (Supplementary Fig. S1, available at *IJE* online). The age-period model was confirmed to be the best-fitting one (data not shown). The linear trend was decomposed into the contribution of the cumulative exposure to screening and a residual linear trend. We estimated a relative increase in incidence of 8.9% (95% CI: 0.0%; 19.0%) every 5-year span of exposure to screening. The residual linear trend was estimated as an increase in incidence of 17.0% (95% CI: 4.9%; 30.4%) every 5-year calendar period.

## Discussion

### Principal findings and interpretation

The APC analysis of time trend in incidence rates of DCIS in this screening-aged population showed that the birth cohort had a 2-fold effect. The nonlinear effect consisted of an increase in incidence for women born in 1933–42 and 1943–52, who have been targeted by the screening programme since 1997, and of a second increase for women born in 1963–72, first invited in 2010. The linear effect consisted of a marked increase in the risk of DCIS for all successive birth cohorts or calendar periods of 4.0% per year, which was only partially attributable to the screening programme. In brief, the observed pronounced increase in incidence rates resulted from the concomitance of an increased detection of DCIS through the screening programme and an uptrend in the risk of diagnosis not attributable to the programme. The latter can be attributed either to long-term changes in diagnostic scrutiny independent of the programme, or to an increased exposure to risk factors, or to a combination of both.

This original finding was made possible by the unusual and complex time-age pattern of implementation of the screening programme in the study area, with the oldest and youngest women being invited more than 10 years after the middle-aged ones. The nonlinear effect of the birth cohort on incidence matched perfectly with the timing of invitation to screening by age group. As a rule, the effect of screening on the incidence of the target disease is visible as a period effect, i.e. as the result of an external factor affecting all age groups. Cohort effects, instead, are variations generally resulting from the unique exposure of a group of subjects over time. The time-age pattern of implementation of mammography screening in the study area, however, caused an interaction between the age group and the calendar period and, thus, a nonlinear cohort effect linked to the screening programme. Interestingly, a comparable situation has been reported from the Nordic Countries in relation to the introduction of cervical cancer screening [33].

### Implications for research

DCIS is considered a nonobligate precursor of invasive BC. At present, however, a great deal of uncertainty still exists about how it develops into an invasive disease and how it can be detected and treated [4]. Current views point to the existence of genetically encoded properties that influence the tumour’s interaction with the immune system and, thus, its aggressiveness, behaviour, ability to progress, and response to treatments [34–36]. In line with this model, our data suggest that starting screening earlier does not seem to prevent DCIS at a later age, and other data suggest that it does not prevent invasive BC at a later age [1, 5, 6].

A better understanding of the natural history of the disease would greatly support patients and healthcare professionals in making informed treatment decisions. Four clinical trials have been undertaken to evaluate the safety of active surveillance for low-risk DCIS, defined as grade I or II and, in one study, oestrogen receptor-positive and HER2-negative [37–41]. The results are expected to provide evidence that part of DCIS is indolent. This would help future patients consider a range of treatment choices [37].

Considering the poor—if any—potential of low-grade DCIS to progress to invasive BC and the tendency of patients to overestimate the risk, it has been proposed to replace the term low-grade DCIS with ductal neoplasia or ductal intraepithelial neoplasia [42, 43]. Pending the results of ongoing clinical trials of active surveillance, a literature review has collated the epidemiologic evidence in favour or against relabelling low-risk DCIS [44]. Overall, studies of natural history, prevalence at autopsy, and diagnostic reproducibility confirm that the removal of ‘cancer’ from the diagnostic label of low-risk DCIS merits consideration. At present, however, this remains a debated choice [45].

The evidence from clinical research should be corroborated with a better understanding of the epidemiologic trend of DCIS in different populations as contrasted to the invasive counterpart. In a future work, we will evaluate—with the due exhaustiveness—the incidence trend of invasive BC in our population. For completeness of information, however, we should anticipate here that the increasing trend of DCIS was not followed by an opposite trend of invasive BC (Supplementary Fig. S4, available at *IJE* online).

### Methodological considerations

There are some methodological issues in the study. First, the observed age pattern of incidence trend should be considered with caution. Previous studies have reported varying findings [1, 5–7, 13–15]. We restricted our analysis to a relatively narrow age group. The increase was distributed across all age subgroups but was more pronounced between 65 and 74 years. This conveys a greater risk of overdiagnosis and overtreatment [5].

Second, studies on the descriptive epidemiology of DCIS have to face the problem of under-registration of the disease, which complicates the understanding of the effects of screening [46]. DCIS was generally not registered until the 1980s at the global level and, subsequently, the IARC rules have inevitably caused an underestimate of incidence rates. Considering this problem, we took the incidence data from our ‘service’ database, which retains all information collected since 1990, including notifications of an *in situ* cancer followed by an invasive cancer. We are confident that this process was accurate. This fact notwithstanding, the past situation may have continued to adversely influence the registration process, thus contributing to the linear increase attributable to the screening programme. Careful consideration of this and other external factors affecting DCIS rates (especially the criteria for pathologic diagnosis and the sensitivity of breast imaging technologies) [10] remains necessary in interpreting our results.

## Conclusions

The increase in incidence rates of DCIS among screening-aged women living in northern Italy resulted from an increased detection of the disease through the screening programme and from an uptrend in the risk of diagnosis independent of the programme. The latter may represent a long-term increase in diagnostic scrutiny and detection (e.g. from increasingly sensitive breast imaging technologies), or a true increase due to increased exposure to risk factors, or a combination of both.

## Ethics approval

The study was approved by the ‘Comitato Etico della Romagna’ (C.E.Rom.), i.e. the ethics committee at the IRCCS Istituto Romagnolo per lo Studio dei Tumori (IRST) Dino Amadori, Meldola, Forlì, Italy (ID: IRST100.37; date of approval: 22 July 2022; IRST identifier code: L1P2509). The C.E.Rom. waived the requirement of informed consent form for this study due to its retrospective nature and because the analysis was an audit using anonymous and routinely collected clinical data.

## Supplementary Material

dyaf102_Supplementary_Data

## Data Availability

The dataset generated for this study is available from the corresponding author upon request.
