# Supplementary material for: Mammography screening and incidence of ductal carcinoma in situ of the breast in Italy: an age-period-cohort analysis
Source: Int J Epidemiol. 2025 Jun 24;54(4):dyaf102. doi: 10.1093/ije/dyaf102 (PMC12188216; doi:10.1093/ije/dyaf102)
Supplement: dyaf102_Supplementary_Data [file dyaf102_supplementary_data.pdf]

International Journal of Epidemiology

Mammography screening and incidence of ductal carcinoma in situ of the breast in Italy: an age-period-cohort analysis

Lauro Bucchi,[1] Silvia Mancini,[1,*] Annibale Biggeri,[2] Rosa Vattiato,[1] Orietta Giuliani,[1] Alessandra Ravaioli,[1] Flavia Baldacchini,[1] Federica Zamagni[1] and Fabio Falcini[1,3]

[1]Emilia-Romagna Cancer Registry, Romagna Cancer Institute, IRCCS Istituto Romagnolo per lo Studio dei Tumori (IRST) Dino Amadori, Meldola, Forlì, Italy, [2]Unit of Biostatistics, Epidemiology and Public Health, Department of Cardiac, Thoracic, Vascular Sciences and Public Health, University of Padua, Padua, Italy, [3]Cancer Prevention Unit, Local Health Authority, Forlì, Italy

*Corresponding author. Emilia-Romagna Cancer Registry, Romagna Cancer Institute, IRCCS Istituto Romagnolo per lo Studio dei Tumori (IRST) Dino Amadori, Meldola, Forlì, Italy. E-mail: silvia.mancini@irst.emr.ita

Supplementary data

Supplementary Figure S1 p. 2
Supplementary Figure S2 p. 3
Supplementary Figure S3 p. 4
Supplementary Table S1 p. 5
Supplementary Table S2 p. 6
Supplementary Figure S4 p. 7

Age group	Calendar period				
	**1992-1996**	**1997-2001**	**2002-2006**	**2007-2011**	**2012-2017**
45-49	0	0	0	1	1
50-54	0	1	1	1	2
55-59	0	1	2	2	2
60-64	0	1	2	3	3
65-69	0	1	2	3	4
70-74	0	0	1*	3	4

0 = no exposure to screening

1 = exposure to screening at i^{th} age group and j^{th} period only

1* = exposure to screening at $(i\text{-}1)^{th}$ age group $(j\text{-}1)^{th}$ period and NOT exposed at i^{th} age group j^{th} period

2 = exposure to screening at i^{th} age group j^{th} period and $(i\text{-}1)^{th}$ age group $(j\text{-}1)^{th}$ period

3 = exposure to screening at i^{th} age group j^{th} period and $(i\text{-}1)^{th}$ age group $(j\text{-}1)^{th}$ period and $(i\text{-}2)^{th}$ age group $(j\text{-}2)^{th}$ period

4 = exposure to screening at i^{th} age group j^{th} period and $(i\text{-}1)^{th}$ age group $(j\text{-}1)^{th}$ period and $(i\text{-}2)^{th}$ age group $(j\text{-}2)^{th}$ period and $(i\text{-}3)^{th}$ age group $(j\text{-}3)^{th}$ period

Supplementary Figure S1. Lexis diagram showing the five-year calendar periods (six years for the period 2012-2017) and the five-year age groups. The cells highlighted in yellow indicate exposure to the screening programme. The numbers denote the degree of cumulative exposure to the screening programme. Romagna (northern Italy), 1992-2017

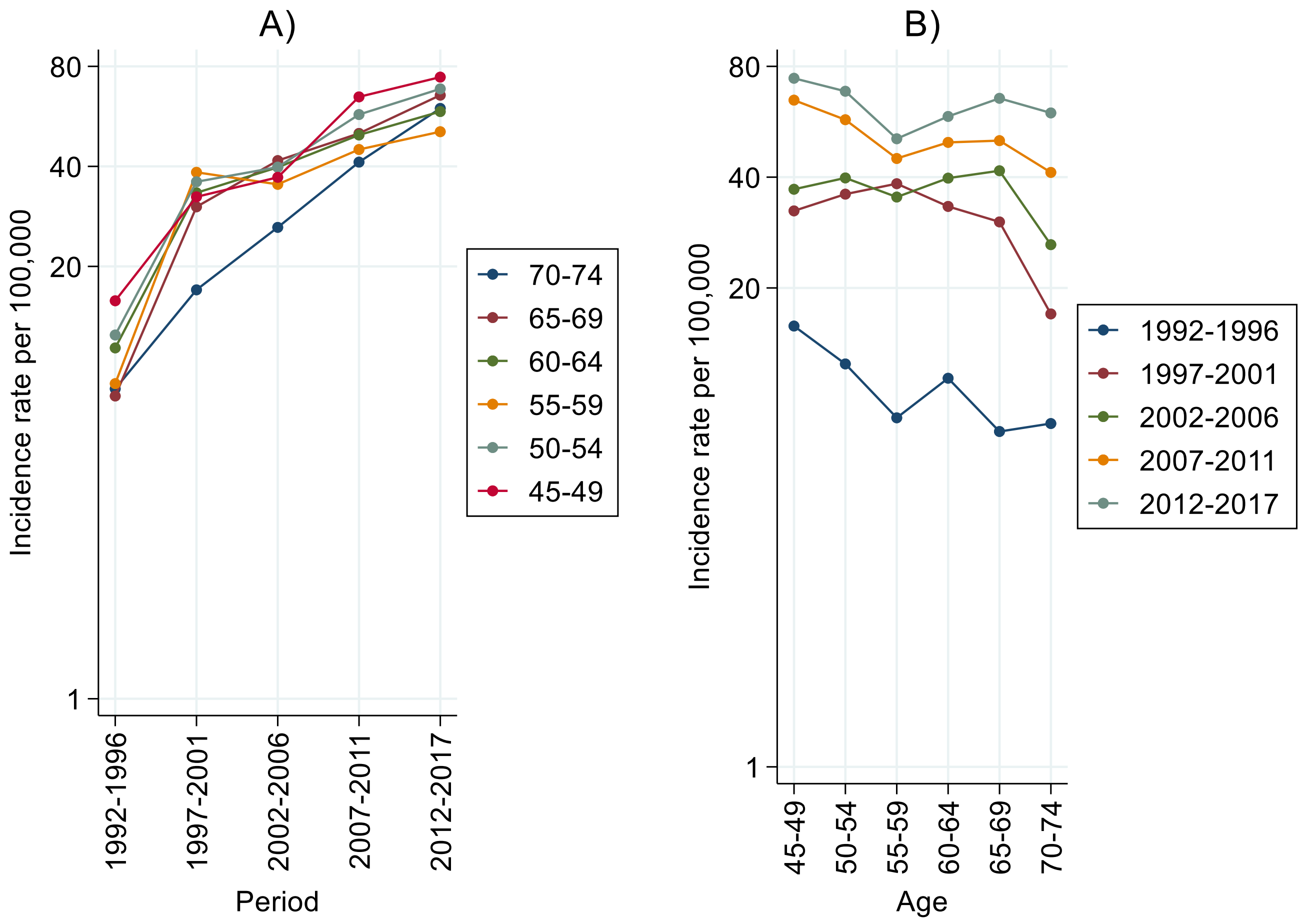

Supplementary Figure S2. A) Plots of five-year calendar period-specific (six years for the period 2012-2017) incidence rates of ductal carcinoma in situ of the breast per 100,000 women, on a semi-logarithmic scale, by five-year age groups. **B)** Plots of five-year age group-specific incidence rates of ductal carcinoma in situ of the breast per 100,000 women, on a semi-logarithmic scale, by five-year calendar periods (six years for the period 2012-2017). Romagna (northern Italy), 1992-2017

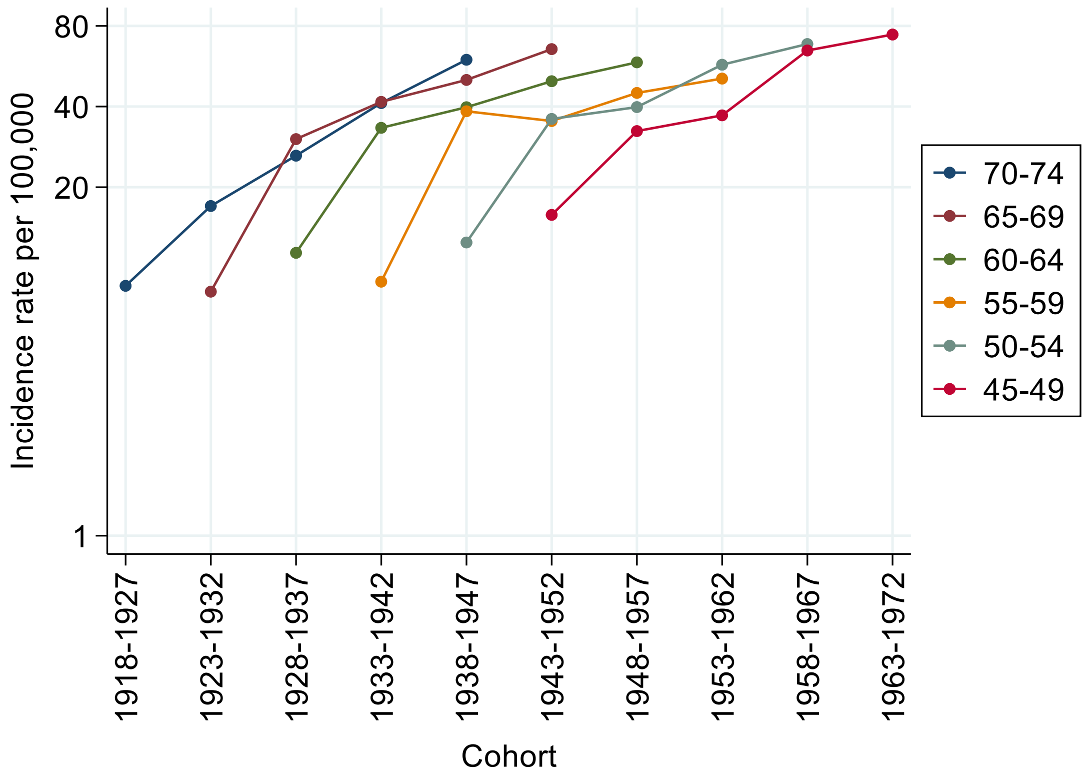

Supplementary Figure S3. Plots of 10-year birth cohort-specific incidence rates of ductal carcinoma in situ of the breast per 100,000 women, on a semi-logarithmic scale, by five-year age groups. Romagna (northern Italy), 1992-2017

Supplementary Table S1. Comparison of age-period-cohort models for the trend in incidence of ductal carcinoma in situ of the breast among women aged 45-74. Romagna (northern Italy), 1992-2017

Terms in the model[a]	Degrees of freedom	Deviance	AIC	Models to compare	Deviance difference	Degree of freedom difference	P-value[b]
1. Age (A)	24	520.2	23.703				
2. Age-drift (Ad)	23	78.9	9.057	Ad versus A	441.4	1	<0.001
3. Age-period (AP)	20	25.5	7.478	AP versus Ad	53.4	3	<0.001
4. Age-cohort (AC)	15	56.1	8.831	AC versus Ad	22.8	8	0.004
**5. Age-period-cohort (APC)**	**12**	**8.8**	**7.456**	**APC versus AP**	**16.7**	**8**	**0.034**
				**APC versus AC**	**47.2**	**3**	**<0.001**

AIC, Akaike information criterion.

[a]Five submodels were derived, namely: age, age-drift, age-cohort, age-period and the full age-period-cohort model. The drift term is a linear temporal variation of rates that cannot be attributed either to the period effect or to the cohort effect. The model goodness-of-fit was tested using residual deviance statistics. The age, period and birth cohort effects were derived from pairwise comparisons of the appropriate submodels. The statistical significance of the pairwise comparisons was tested by comparing the difference in residual deviance and degrees of freedom using the likelihood ratio test. The models 3 and 4 could not be compared directly in this way nor was it possible to perform a formal test of whether the age-cohort model was significantly better than the age-period model. The full age-period-cohort model provided the best fit to the data, as confirmed by the observation that this model minimised the AIC.

[b]Likelihood ratio test.

Supplementary Table S2. Comparison of age-period-cohort models, with a dummy variable for the presence/absence of the screening programme, for the trend in incidence of ductal carcinoma in situ of the breast among women aged 45-74. Romagna (northern Italy), 1992-2017

Terms in the model[a]	Degrees of freedom	Deviance	AIC	Models to compare	Deviance difference	Degree of freedom difference	P-value*
1. Age + screening (As)	23	140.6	11.114				
2. Age-drift + screening (Ads)	22	35.4	7.676	Ads versus As	105.1	1	<0.001
**3. Age-period + screening (APs)**	**19**	**12.8**	**7.121**	**APs versus Ads**	**22.6**	**3**	**<0.001**
4. Age-cohort + screening (ACs)	14	24.8	7.855	ACs versus Ads	10.6	8	0.224
5. Age-period-cohort + screening (APCs)	11	5.3	7.405	APCs versus APs	7.5	8	0.484
				APCs versus ACs	19.5	3	<0.001

AIC, Akaike information criterion.

[a]Five submodels were derived, namely: age, age-drift, age-cohort, age-period and the full age-period-cohort model, each with a term for the presence/absence of the screening programme. The drift term is a linear temporal variation of rates that cannot be attributed either to the period effect or to the cohort effect. The model goodness-of-fit was tested using residual deviance statistics. The age, period and birth cohort effects were derived from pairwise comparisons of the appropriate submodels. The statistical significance of the pairwise comparisons was tested by comparing the difference in residual deviance and degrees of freedom using the likelihood ratio test. The models 3 and 4 could not be compared directly in this way nor was it possible to perform a formal test of whether the age-cohort model was significantly better than the age-period model. The age-period model with a term for the presence/absence of the screening programme provided the best fit to the data, as confirmed by the observation that this model minimised the AIC.

[b]Likelihood ratio test.

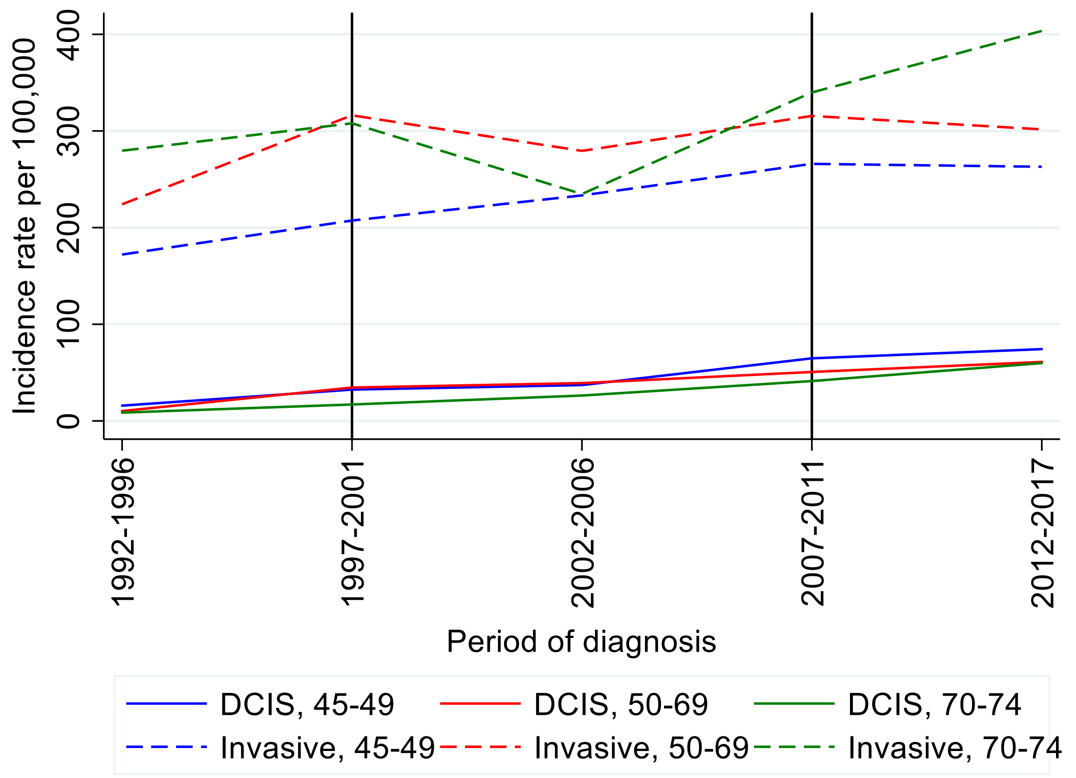

Supplementary Figure S4. Curves of five-year calendar period-specific (six years for the period 2012-2017) incidence rates of ductal carcinoma in situ of the breast and invasive breast cancer per 100,000 women, by age groups. The vertical lines indicate the calendar periods during which the first round of the screening programme was initiated (i.e., 1997-2001 for women aged 50-69 and 2007-2011 for women aged 45-49 and 70-74). Romagna (northern Italy), 1992-2017
